# Leaching Behaviors of Chromium(III) and Ammonium-Nitrogen from a Tannery Sludge in North China: Comparison of Batch and Column Investigations

**DOI:** 10.3390/ijerph17166003

**Published:** 2020-08-18

**Authors:** Xiangke Kong, Yanyan Wang, Lisha Ma, Guoxin Huang, Zhaoji Zhang, Zhantao Han

**Affiliations:** 1 Institute of Hydrogeology & Environmental Geology, Chinese Academy of Geological Sciences, Shijiazhuang 050061, China; kongxiangke@mail.cgs.gov.cn (X.K.); wangyanyan@mail.cgs.gov.cn (Y.W.); malisha@mail.cgs.gov.cn (L.M.); zhangzhaoji@mail.cgs.gov.cn (Z.Z.); 2 Key Laboratory of Groundwater Remediation of Hebei Province and China Geological Survey, Shijiazhuang 050061, China; 3 Chinese Academy for Environmental Planning, Beijing 100012, China; huanggx@caep.org.cn; 4 Technical Centre for Soil, Agriculture and Rural Ecology and Environment, Ministry of Ecology and Environment, Beijing 100012, China

**Keywords:** tannery sludge, trivalent chromium, ammonium-nitrogen, leaching behavior

## Abstract

Tannery sludge usually has high content of trivalent chromium (Cr(III)) and ammonium-nitrogen (NH_4_^+^-N). It is important to make a critical evaluation of the releasing behaviors of Cr(III) and NH_4_^+^-N from tannery sludge before its use on improving soil fertility in agricultural applications. For this purpose, static batch and dynamic leaching experiments with different mathematical models were carried out to simulate the Cr(III) and NH_4_^+^-N releasing kinetics from tannery sludge sampled in a typical tannery disposal site in North China, and their influencing factors were also discussed. The results showed that a larger solid-liquid ratio, a higher temperature, and a lower pH value of the leaching solution were beneficial for the release of Cr(III) and NH_4_^+^-N from the tannery sludge. The release kinetics of Cr(III) and NH_4_^+^-N followed parabolic diffusion and simple Elovich models both in the static and dynamic leaching conditions, indicating that the release was a complex heterogeneous diffusion process. The NH_4_^+^-N was easy to be leached out and its released amount reached 3.14 mg/g under the dynamic leaching condition (pH 7), whereas the released amount of the Cr(III) was only 0.27 μg/g from the tannery sludge. There was a positive correlation coefficient between dissolved Fe and Cr(III) in the leachate under different leaching conditions, and the calculated average ratio of Fe/Cr(III) concentration was 3.56, indicating that the small amount of the released Cr(III) came from the dissolution of Cr_0.25_Fe_0.75_(OH)_3_ minerals in tannery sludge.

## 1. Instruction

In China, large quantities of tannery sludge (about 30 million tons per year), which usually contains a high content of trivalent chromium (Cr(III)) (1–4%, wt/%), are generated during the tanning process [1,2]. However, due to the lack of landfill sites and expensive treatment costs, the illegal disposal of tannery sludge onto farmland by some tanning factories was a common phenomenon over the past few years in China [3]. The release risk and accumulation of Cr(III) from tannery sludge has become a major environmental concern [4,5].

Cr(III) is relatively less toxic and stable when the soil pH is above 6.0 [6]. Therefore, for the tannery sludge which were pretreated by iron flocculation and precipitation, some researchers suggested that applying them in agricultural soil can effectively solve their disposal problem in the environment [7]. However, during the long-term leaching condition, there still exists a high release risk of Cr(III) from this kind of tannery sludge, which often contains a high Cr(III) content of tens of milligrams per gram [8]. In addition, the accumulation of Cr(III) in soil and water also have a serious toxic effect on human health and the environment, which can cause allergic skin reactions and cancer [9,10]. The World Health Organization and the US Environmental Protection Agency have set the maximum permissible concentration of Cr in drinking water at 50 μg/L [11]. The releasing rate and cumulative release amount of Cr(III) from tannery sludge in different leaching conditions needs to be analyzed carefully before its agricultural applications, especially in the acid rain region [12]. In addition to Cr(III), tannery sludge also contains a high content of ammonium-nitrogen (NH_4_^+^-N) due to the abundant use of ammonium salt in the deliming process. Although an appropriate content of NH_4_^+^-N would have some beneficial effects on soil fertility, whereas a high concentration of NH_4_^+^-N releasing from tannery sludge may cause the nitrogen accumulation in soils. Under the long-term action of indigenous nitrifying bacteria, NH_4_^+^-N is easily transformed in the soil to nitrate (NO_3_^−^-N) [13], causing the secondary contamination to deep soils and groundwater. New investigation of a typical soil profile with long-term tannery sludge contamination demonstrated that the characteristic contaminants were Cr(III) and NH_4_^+^-N in shallow soil [14]. Nevertheless, limited efforts have been directed towards the assessment of Cr(III) and NH_4_^+^-N release behaviors from the tannery sludge under different leaching conditions.

This paper focuses primarily on the release characteristics and mechanisms of Cr(III) and NH_4_^+^-N from the tannery sludge. The static batch and dynamic leaching experiments were conducted to evaluate the release behaviors of Cr(III) and NH_4_^+^-N under the different leaching conditions. In addition, the factors influencing the release mechanisms of Cr(III) and NH_4_^+^-N, such as the contact time, liquid-solid ratio, temperature, and pH of the leaching solution, were explored using different mathematical models (parabolic diffusion, power function and simple Elovich). The corresponding achievements were expected to provide a scientific basis to support the pollution risk assessment and effective disposal of the tannery sludge.

## 2. Materials and Methods

### 2.1. Tannery Sludge and Its Characterization

The tannery sludge used in the experiments was obtained from an illegal tannery sludge disposal site in Hebei province of China (37°52′ N, 115°15′ E), which was mainly generated from the neighboring tannery industries. The sampling was repeated five times at the different storage points of tannery sludge to obtain five subsamples (1 kg). Subsequently, a representative sample (5 kg) was taken by mixing them well at each sampling point, and then directly placed in a pre-cleaned polyethylene sealable bag. On return to the laboratory, the tannery sludge was air-dried, crushed, homogenized, and passed through 20 mesh nylon sieves for the subsequent batch and column experiments.

The physicochemical characteristics of the tannery sludge were shown in Table 1. The alkaline tannery sludge has high contents of moisture and organic matter. Due to the simple pretreatment by ferric flocculant and precipitation with alkali before sludge dumping, the high content of Cr(III) (30,800 mg/kg) mainly existed in a stable state of Fe/Cr oxides in the tannery sludge, which was demonstrated by the X-ray diffraction (XRD), X-ray fluorescence (XRF) and X-ray photoelectron spectroscopy (XPS) results in our previous study [14]. The NH_4_^+^-N (16,080 mg/kg) and organic-nitrogen (16,500 mg/kg) were extremely high in the tannery sludge, which were generated during the tanning processes of NH_4_^+^-N deliming and protein catabolism [15].

### 2.2. Experimental Set-Up

#### 2.2.1. Batch Leaching Experiments

Three batch experiments were designed to evaluate the effects of solid-liquid ratio (SLR), temperature, and pH on the release of Cr(III) and NH_4_^+^-N from tannery sludge to solution. For the SLR experiments, 1 g of the tannery sludge samples were respectively added into a series of 50 mL centrifuge tubes and a certain volume of deionized water was added to keep the SLR ratio to be 1:10, 1:20 and 1:40. The tubes were shaken in a shaker (170 rpm) at room temperature (25 °C). For the temperature experiments, 1 g of the tannery sludge samples were added into a series of 50 mL centrifuge tubes with 10 mL deionized water. The tubes were shaken in a shaker (170 rpm) at 15 °C, 25 °C, and 35 °C, respectively. For the pH experiments, 1 g of the tannery sludge were mixed with 10 mL solution with different pH value in a series of 50 mL centrifuge tubes, respectively. The initial solution pH was adjusted to 1.0, 3.0, 5.0, and 7.0 by HCl or NaOH solution. The tubes were shaken in a shaker (170 rpm) at room temperature (25 °C). All the experiments were carried out in duplicate. The redox potential (ORP) and pH of solutions were measured immediately, and the samples were collected and filtered through a 0.45-μm membrane filter prior to the measurement of total Cr, Fe, Cr(III), and NH_4_^+^-N concentrations. Unless otherwise indicated, all chemicals and reagents used in the experiments were analytical grade.

#### 2.2.2. Column Leaching Experiment

Effect of acid rain pH on leaching behavior of Cr(III) and NH_4_^+^-N from tannery sludge were simulated. The column leaching experiments were conducted with three glass columns (height, 10 cm; internal diameter, 2.4 cm) to evaluate the dynamic release characteristics and the cumulative release amounts of Cr(III) and NH_4_^+^-N with different pH leaching solution. The middle parts of the columns were packed with dry tannery sludge (30 g), and a layer (1 cm and 1.5 cm) of fine quartz sand (40–80 mesh) were respectively placed on the top and bottom of the columns to ensure a good flow distribution. The leaching solution with different pH values (adjusted to 3.0, 5.0, and 7.0 by HCl) were pumped downward through the three columns by a multi-channel peristaltic pump, respectively. The leaching rate was set as 62.5 mL/d based on the infiltration rate of precipitation in the study area. The leachate samples were collected at regular interval of time and filtered immediately through a 0.45-μm membrane filter prior to the measurement of the total Cr, total Fe, Cr(III), Cr(VI), and NH_4_^+^-N concentrations.

### 2.3. Analytical Methods

The total Cr and Fe concentrations of leachate were determined by inductively coupled plasma atomic emission spectrometry (iCAP6300, Thermo Scientific, Waltham, MA, USA). The Cr(VI) concentration was determined with 1,5-diphenylcarbazide at 540 nm, using a UV-visible spectrophotometer (UV-2550, Shimadzu, Kyoto, JPN). The Cr(III) concentration was calculated by subtracting Cr(VI) from the total Cr concentration. The NH_4_^+^-N and NO_3_^−^-N were analyzed using the UV-2550 spectrophotometer at λ_max_ 420 nm, 220 nm and 275 nm, respectively. The concentrations of dissolved organic matter (DOC) and organic nitrogen were determined by a total organic carbon analyzer (TOC-L CPH, Shimadzu, Kyoto, JPN). An elemental analyzer (vario EL cube, Elementar, Frankfurt, GER) was utilized for the element analyses of C and N. Solution pH was measured with a digital pH meter (PB-10, Sartorius, Goettingen, Germany). The ORP value of solution was determined using a portable ORP analyzer (A221, Thermo Orion, Waltham, MA, USA). The instruments were all calibrated before measuring the samples. The minimum detectable concentration of total Cr, Fe, Cr(VI), NH_4_^+^-N, and NO_3_^−^-N were 0.05 mg/L, 0.04 mg/L, 0.004 mg/L, 0.025 mg/L and 0.08 mg/L, respectively.

### 2.4. Mathematical Models

Previous studies have demonstrated that the release kinetics of inorganic cations and heavy metals from sludge can be simulated more accurately by the parabolic diffusion (Equation (1)), power function (Equation (2)) and simple Elovich (Equation (3)) models [16,17]. The three classic models above were used to characterize the release kinetics of Cr(III) and NH_4_^+^-N from the tannery sludge, which were described as follows.

(1) $$\mathrm{Parabolic}\;\mathrm{diffusion}:\;q_{t}=a+k_{p}t^{1/2}$$

(2) $$\mathrm{Power}\;\mathrm{function}:\;\ln q_{t}=\ln b+k_{f}lnt$$

(3) $$\mathrm{simple}\;\mathrm{Elovich}:\;q_{t}=c+k_{s}lnt$$

where $q_{t}$ (mg·kg^−1^, dry weight) is the amount of compounds released after reaction time (h); a, b, and c are the constant; *t* is the time (*h*); $k_{p}$ is the diffusion rate constant (mg·kg^−1^)^−0.5^; $k_{f}$ is the rate coefficient value (mg·kg^−1^)^−1^; $k_{s}$ is the release rate constant (mg·kg^−1^). All parameters can be determined by the regression of the experimental data which were fitted using the Origin 8.0 software (OriginLab, Northampton, MA, USA).

The total release amount ($q_{total}$, mg) of Cr(III) or NH_4_^+^-N in each column is equal to the area under the plot of the effluent concentration (*C_t_*, mg/L) versus leaching time at the condition of a given flow rate (Equation (4)).

(4) $$q_{total}=\frac{Q}{1000}\int_{t=0}^{t=t_{total}}C_{t}d_{t}$$

where *Q* is the leaching volume (mL) and *t_total_* is the total leaching time (h) of each column, respectively.

The average release amount (*q_av_*, mg/g) of Cr(III) and NH_4_^+^-N from the tannery sludge can be further calculated using *q_total_* to divide the weight of the tannery sludge in each column (Equation (5)):

(5) $$\;q_{av}=\frac{q_{total}}{m}$$

where *m* is the dry mass of the tannery sludge filled in each column (g).

## 3. Results and Discussion

### 3.1. Releasing Behavior of Cr(III) and NH_4_^+^-N under the Different Experimental Conditions

**Figure 1:**
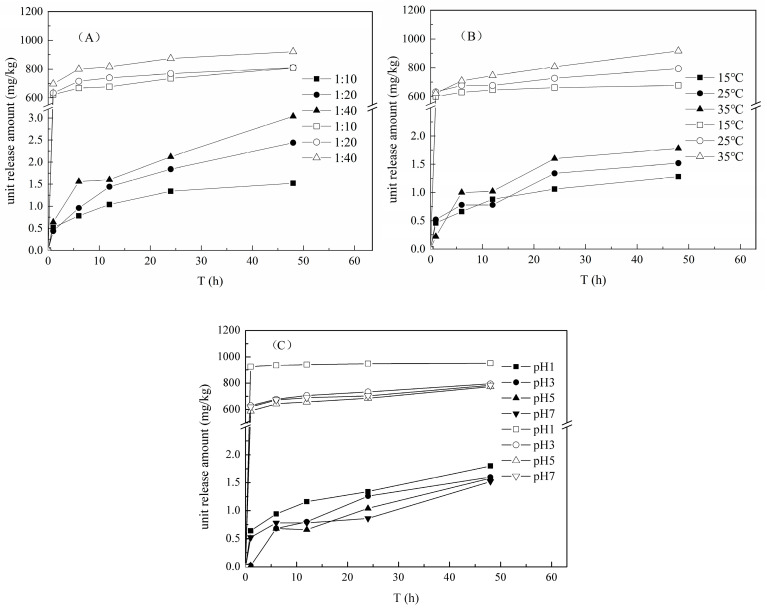
The release amounts of Cr(III) and NH_4_^+^-N under the different conditions ((**A**): solid-liquid ratio (SLR); (**B**): temperature; (**C**): pH; Solid legend means Cr(III), and hollow legend means NH_4_^+^-N).

The release curves of Cr(III) and NH_4_^+^-N from the tannery sludge under the conditions of different SLR, temperature and pH values were shown in Figure 1. The unit release amounts of Cr(III) and NH_4_^+^-N both increased steadily with the increasing contact time under the different reaction conditions, and the release processes of Cr(III) and NH_4_^+^-N were both composed of two steps of different mechanisms: rapid release and slow release. At the initial rapid release phase (0–1 h), the released Cr(III) and NH_4_^+^-N mainly came from the water-soluble fraction in the pore structure and the insoluble fraction adsorbed through van der Waals forces on the surface of tannery sludge, which were easily desorbed and released into the aqueous phase from the solid medium [18,19]. At the subsequent slow release phase (1–24 h), the release rates of Cr(III) and NH_4_^+^-N decreased drastically and became stabilized after 24 h. This could be attributed to the release of these cationic fractions strongly adsorbed on the mineral and internal surfaces in the tannery sludge [20,21]. Under the condition of SLR 1:10, T 25 °C and pH 7, the maximum unit release amount of NH_4_^+^-N reached to 808.5 mg/kg, whereas the maximum release amount of Cr(III) was only 1.5 mg/kg. The release amount of NH_4_^+^-N was significantly larger and the release rate was faster compared to Cr(III). Besides, no Cr(VI) (below detectable limit, 0.004 mg/L) was detected under the different experimental conditions.

With the increase of SLR value, the unit release amounts of Cr(III) and NH_4_^+^-N increased remarkably (Figure 1A). There would be enough water to react with the tannery sludge at a high SLR value. On the other hand, the mass transfer resistance could be decreased in the water-solid interface at a high SLR value, which could increase the diffusion ability of dissolved compounds [22].

**Figure 2:**
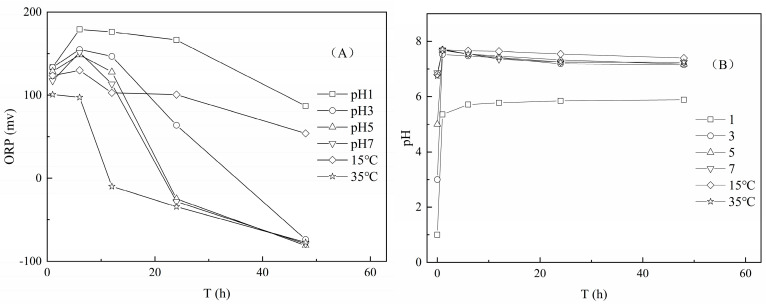
The Changes of ORP and pH values in leachate under the different conditions ((**A**): redox potential (ORP); (**B**): pH).

Generally, the diffusion capacity of ions is promoted at a high temperature in the solid-liquid system [23,24], and an elevated temperature increased the unit release amounts of Cr(III) and NH_4_^+^-N from tannery sludge (Figure 1B). Since the tannery sludge contains high content of nitrogenous organic matters, such as organic acid, fat, and protein, it is easier for the organic matter decomposition by microbial activity [25]. The ORP value of solution decreased rapidly from initial 130 mv to nearly −70 mv at the relatively high temperature and neutral pH condition (Figure 2A), which indicated the occurrence of the organic matter decomposition of the tannery sludge. Under the reduction condition, the released NH_4_^+^-N was increased due to the hydrolysis and decomposition of nitrogenous organic matters in tannery sludge [8]. In addition, the iron (Fe) bioreduction would be promoted with the organic matter mineralization [26], and partial Cr(III) combined with Fe oxides could be released into solution with the dissolution of Fe complex in the tannery sludge.

The unit release amount of Cr(III) increased with the decrease in pH, whereas the release amount of NH_4_^+^-N was obviously increased only at the extremely acidic condition (pH 1) (Figure 1C). The dissolution and desorption processes of ions from sediments were promoted at lower pH value [22]. Clearly, the high H^+^ concentration of solution promoted the ion-exchange of H^+^ with cationic Cr(III) and NH_4_^+^-N in the tannery sludge. However, at the condition of initial pH range of 3.0–7.0, the pH all increased rapidly to above 7.2, correspondingly (Figure 2B). This could be attributed to the strong acid buffer capacity of the tannery sludge, which limited the desorption and release of Cr(III) and NH_4_^+^-N. 

In summary, the SLR, temperature and pH of the leachate were found to influence the release processes of Cr(III) and NH_4_^+^-N. A larger SLR, a higher temperature, and a lower pH value of the leachate are beneficial for the Cr(III) and NH_4_^+^-N release from the tannery sludge to solution.

### 3.2. Release Kinetics Simulated by Mathematical Models

As shown in Table 2, the Cr(III) and NH_4_^+^-N release processes are fitted well with the parabolic diffusion, power function and simple Elovich models, as indicated by *R*^2^ values of 0.857–0.999 (NH_4_^+^-N) and 0.799–0.944 (Cr(III)), respectively. These diffusion models were used to indicate a diffusion-controlled phenomena of the ion release from soils and medium [27,28]. Thus, the good fitting results suggested that the Cr(III) and NH_4_^+^-N release from the tannery sludge was mainly a heterogeneous diffusion-controlled release process. The release rate constants of $k_{p}$ and $k_{s}$ were much higher for NH_4_^+^-N than those for Cr(III) under the same condition. This was possibly due to the higher dissolution and desorption abilities of NH_4_^+^-N than those of Cr(III) in solution. Moreover, the constants $k_{p}$ and $k_{s}$ for NH_4_^+^-N in different models had a positive correlation with SLR and temperature, whereas no significant relationship was found between these constants and pH. The values of $k_{p}$ and $k_{s}$ increased with increasing in SLR and temperature, which further demonstrated that the NH_4_^+^-N release was mainly controlled by diffusion-driven dissolution process [29]. In contrast, The Cr(III) species mainly precipitated as Cr(OH)_3_(s) and (Fe,Cr)(OH)_3_(s) in the tannery sludge, and the release of Cr(III) was appeared to be controlled by a solubility process instead of an adsorption-desorption reaction [30,31]. The values of $k_{p}$, $k_{f}$, and $k_{s}$ for Cr(III) in different models all increased with the decrease of pH, suggesting that the release of Cr(III) was controlled by the dissolution-precipitation process.

**Figure 3:**
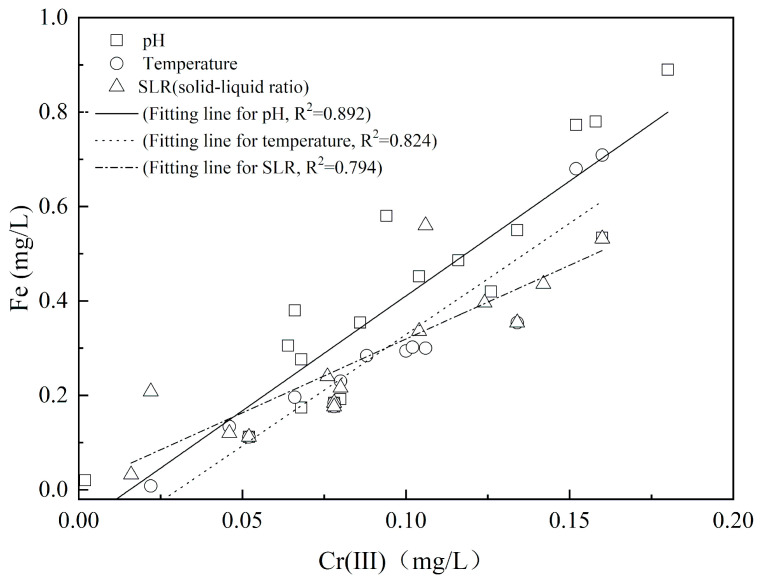
The relationship between dissolved Fe and Cr(III) concentration released into solution under the different leaching conditions.

As shown in Figure 3, there was a positive correlation coefficient between the concentration of dissolved Fe and Cr(III) released into solution under the different pH (R^2^ = 0.892), temperature (R^2^ = 0.824) and SLR (R^2^ = 0.794) conditions. The calculated average ratio of dissolved Fe/Cr(III) concentration was 3.56, which was rather close to the value 3.0 [32] and 3.33 [33] of Fe/Cr content in the precipitation pattern of Cr_0.25_Fe_0.75_(OH)_3_ reported by the literature. Besides, the XRD, XRF, and XPS results have demonstrated the Cr(III) was immobilized as a stable Fe/Cr co-precipitation state in the tannery sludge in our previous study [14]. Thus, the small release amount of Cr(III) was mainly from the Cr_0.25_Fe_0.75_(OH)_3_ dissolution in the tannery sludge, which was mainly influenced by the low pH and high temperature of leaching solution.

### 3.3. Dynamic Leaching Behavior of Cr(III) and NH_4_^+^-N under Different Leachate pH

**Figure 4:**
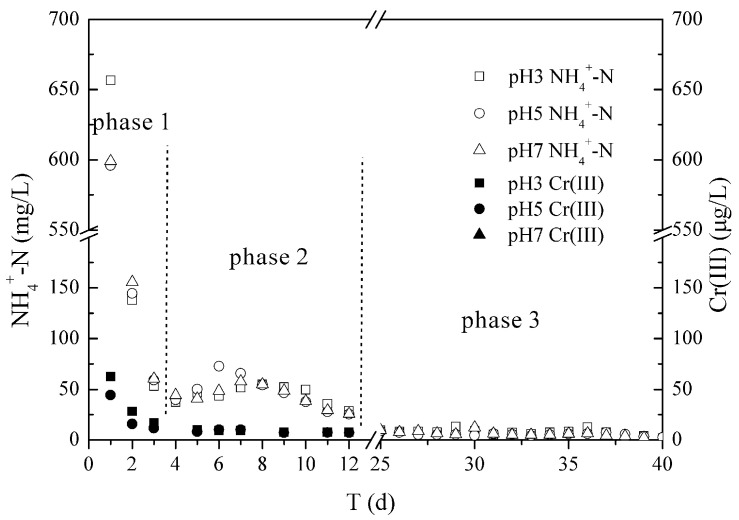
Leaching concentrations of Cr(III) and NH_4_^+^-N under different leachate pH.

As shown in Figure 4, with the increase in leaching volume, the effluent concentrations of Cr(III) and NH_4_^+^-N from the tannery sludge were both decreased under the different leachate pH condition (3.0, 5.0, 7.0). The Cr(III) of below 50 μg/L was only detected in the first 12 days in the three column effluents. The alkaline tannery sludge had a strong acid buffer capacity, as demonstrated by the effluent pH of columns of above 7.12, even at the condition of influent pH 3.0. Thus, the Cr(III) was easily precipitated again and then was difficult to leach out from the columns. In contrast, the NH_4_^+^-N in the effluent were all above 25 mg/L in the first 12 days of leaching, and its release process could be divided into 3 steps. In the initial rapid release phase (0–4 d), the large amount of released NH_4_^+^-N was mainly existed as the soluble forms in the pores, adsorbed on the surface by van der Waals forces and cations exchange in tannery sludge. Meanwhile, a quick decrease of DOC concentration (from a high of 464 mg/L down to 78 mg/L) was also observed in the first 4 days under a different leaching condition, indicating that large amounts of dissolved organic matters were first leached out from the tannery sludge. In the second gradual release phase (5–12 d), the leached NH_4_^+^-N was mainly adsorbed in the inner pores of organic matter and minerals, controlled by the intra-particle diffusion [34]. In addition, owing to the fact that the tannery sludge has a high organic nitrogen content and a low C/N ratio (4.53:1) (Table 1), which is beneficial to the hydrolysis of macromolecular organic nitrogen and occurrence of microbial ammonification [12]. The NH_4_^+^-N was transformed by the decomposition of organic nitrogen in the tannery sludge and its concentration in column effluent dropped slowly during this phase. The leached DOC was also kept at a stable concentration range (from 36.4 to 68.0 mg/L). In the last phase (12–40 d), the small amount of strongly fixed NH_4_^+^-N was gradually desorbed from the tannery sludge. The easily decomposable organic matters in the tannery sludge were almost completely consumed, and only low concentrations of DOC (below 3.6 mg/L) were detected in the effluent of different columns.

**Figure 5:**
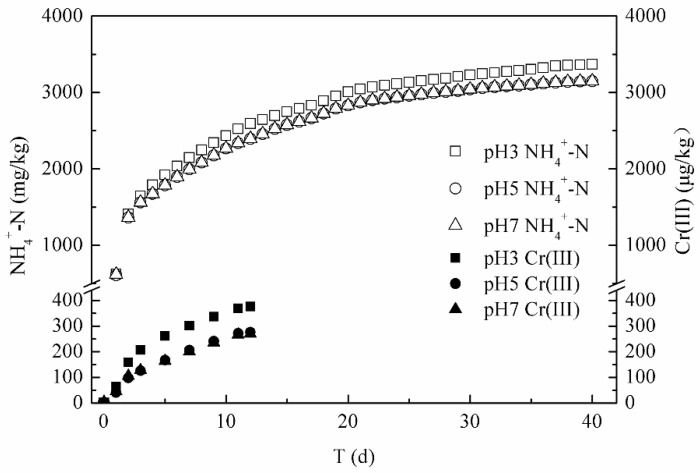
Accumulation releasing amounts of Cr(III) and NH_4_^+^-N from tannery sludge.

The amounts of Cr(III) and NH_4_^+^-N released from the tannery sludge were cumulatively added and plotted against the release time (Equation (4)). As shown in Figure 5, the calculated release amount of Cr(III) was only 0.38 μg/g (pH 3), 0.28 μg/g (pH 5), and 0.27 μg/g (pH 7), while the release amount of NH_4_^+^-N reached to 3.37 mg/g (pH 3), 3.15 mg/g (pH 5), and 3.14 mg/g (pH 7). The release characteristics of Cr(III) and NH_4_^+^-N in column leaching experiments were consistent with the results from the batch experiments, and they all indicated that the high content of NH_4_^+^-N was rather easily released from the tannery sludge. Under the long-term leaching conditions, in addition to the direct contamination from the released high concentration of NH_4_^+^-N to shallow soil, the NO_3_^−^-N transformed by microbial nitrification has a greater pollution risk to deep soil and groundwater.

## 4. Release Kinetics under Leaching Conditions

As shown in Table 3, the leaching release curves of Cr(III) and NH_4_^+^-N were also well fitted by parabolic diffusion and simple Elovich models (*R*^2^ = 0.93–0.98), which further indicated that the releases of Cr(III) and NH_4_^+^-N were controlled by multiple factors such as the adsorption-desorption rate and diffusion rate. There was no significant change in the NH_4_^+^-N release rate constant under the different leachate pH, which was consistent with the results from the bath experiments. In contrast, the Cr(III) release rate constant was significantly affected by the leachate pH. With the leachate pH increasing from 3.0 to 7.0, the $k_{f}$ decreased from 0.53 to 0.44 and the $k_{s}$ decreased from 57.56 to 44.47, respectively. These results further indicated that the acidic leachate promoted the release of Cr(III) from Cr_0.25_Fe_0.75_(OH)_3_ mineral in the tannery sludge, as mentioned in Section 3.2.

## 5. Conclusions

Based on the research findings, the main conclusions were as follows:

1. The release of Cr(III) and NH_4_^+^-N from the tannery sludge to solution was controlled by a series of interactive physical-chemical reactions. Contact time, solid-liquid ratio, temperature, and pH of the leachate were found to significantly influence the Cr(III) and NH_4_^+^-N release process. 

2. Parabolic diffusion and simple Elovich models both can satisfactorily describe the release kinetics of Cr(III) and NH_4_^+^-N from the tannery sludge in the static and dynamic leaching conditions. The NH_4_^+^-N mainly presented as water-soluble and exchangeable fractions in the tannery sludge, and the diffusion-controlled process primarily influenced its release to solution. There was a positive correlation coefficient between Fe/Cr(III) released into solution under the different leaching conditions, and the small amount of Cr(III) released was mainly from the Cr_0.25_Fe_0.75_(OH)_3_ dissolution in the tannery sludge. 

3. The release amount of NH_4_^+^-N reached to 3.14 mg/g under the long-term dynamic leaching condition (pH 7.0), whereas the release amount of Cr(III) was only 0.27 μg/g. Overall, There is low risk of Cr(III) release from the tannery sludge in the natural environment, and more attention should be paid to the large amount of released NH_4_^+^-N from the tannery sludge during the leaching process.

## Figures and Tables

**Table 1:** Physicochemical characteristics of the tannery sludge.

Parameter	Value	Parameter	Value	Parameter	Value
pH	7.67	Total Cr/mg/kg	30,970	Cr_2_O_3_/%	29.00
Moisture/%	64.10	Cr(III)/mg/kg	30,800	Fe_2_O_3_/%	28.61
TOC/wt %	14.30	Cr(VI)/mg/kg	170	CaO/%	7.04
Salinity/mg/kg	99,000	total nitrogen/mg/kg	33,080	SiO_2_/%	5.80
C/%	11.93	NH_4_^+^-N/mg/kg	16,080	Na_2_O/%	3.87
N/%	2.63	organic-nitrogen/mg/kg	16,500	Al_2_O_3_/%	2.19

**Table 2:** Calculated kinetic parameters for Cr(III) and NH_4_^+^-N release from the tannery sludge.

**NH_4_^+^-N**		**Parabolic Diffusion**			**Power Function**			**Simple Elovich**		
		a	$k_{p}$	***R^2^***	b	$k_{f}$	***R^2^***	***c***	$k_{s}$	***R^2^***
SLR	1:10	715.610	53.845	0.999	756.801	0.085	0.962	751.210	77.067	0.937
	1:20	844.190	55.292	0.984	893.816	0.073	0.885	887.540	76.290	0.857
	1:40	707.350	111.890	0.987	787.387	0.146	0.952	783.250	159.340	0.916
T(°C)	15	694.220	54.342	0.998	738.484	0.076	0.968	735.340	64.044	0.955
	25	681.610	92.591	0.994	762.498	0.116	0.959	751.860	85.245	0.886
	35	621.510	165.670	0.991	768.546	0.185	0.979	742.930	154.310	0.904
pH	1	1018.100	16.480	0.910	1028.236	0.020	0.991	1028.100	20.738	0.992
	3	724.350	72.013	0.984	789.895	0.088	0.905	783.900	82.109	0.882
	5	721.610	66.476	0.971	773.557	0.089	0.968	769.720	79.520	0.957
**Cr(III)**		**Parabolic Diffusion**			**Power Function**			**Simple Elovich**		
		a	$k_{p}$	***R^2^***	b	$k_{f}$	***R^2^***	***c***	$k_{s}$	***R^2^***
SLR	1:10	0.349	0.140	0.988	0.496	0.234	0.946	0.457	0.194	0.871
	1:20	0.261	0.277	0.969	0.455	0.408	0.989	0.408	0.412	0.980
	1:40	0.367	0.209	0.971	0.602	0.264	0.923	0.535	0.287	0.843
T(°C)	15	0.299	0.158	0.990	−0.807	0.264	0.978	0.420	0.185	0.938
	25	0.281	0.204	0.965	−0.697	0.278	0.945	0.453	0.181	0.923
	35	0.368	0.243	0.977	−0.499	0.283	0.970	0.556	0.222	0.858
pH	1	0.379	0.290	0.989	0.640	0.362	0.994	0.594	0.344	0.958
	3	0.223	0.288	0.972	0.517	0.338	0.944	0.452	0.323	0.882
	5	0.090	0.281	0.948	0.433	0.318	0.898	0.347	0.318	0.799

“SLR” means the solid-liquid ratio.

**Table 3:** Kinetic model fitting parameters for Cr(III) and NH_4_^+^-N released from tannery sludge.

**NH_4_^+^-N**		**Parabolic Diffusion**			**Simple Elovich**		
		b	$k_{f}$	***R^2^***	***c***	$k_{s}$	***R^2^***
pH	3.0	6.76	0.27	0.99	763.56	372.52	0.97
	5.0	6.66	0.29	0.99	674.55	381.30	0.97
	7.0	6.65	0.29	0.99	662.17	370.36	0.96
**Cr(III)**		**Parabolic Diffusion**			**Simple Elovich**		
		b	$k_{f}$	***R^2^***	***c***	$k_{s}$	***R^2^***
pH	3.0	4.07	0.53	0.96	50.81	57.56	0.98
	5.0	3.90	0.48	0.98	48.00	43.00	0.93
	7.0	4.03	0.44	0.98	40.12	44.74	0.93
